# Hospital Dietetics

**Published:** 1916-12-09

**Authors:** 


### The Hospital

The Workers' Newspaper of Administrative Medicine and Institutional Life, Administration, National Insurance and Health.

No. 1592, Vol. LXI. SATURDAY, DECEMBER 9, 1916. PRICE ONE PENNY

### HOSPITAL DIETETICS.

On another page we print the second part of a paper on this subject by an American physician. It would seem from this lecture (the first portion of which appeared in our columns last week) that in America there are special dietetic officers attached to the hospitals, but what the functions of these officers are remains obscure. In this country a physician would have something to say to a dietetic officer who should prescribe or regulate the diet of one of the physician's patients; and what he would say would be worth hearing. There are some diseases, such as diabetes, typhoid fever, and rickets, in which the regulation of the diet is the chief mode of treatment; and there are others, such as certain forms of dyspepsia, of constipation, and of headache, in which it is important that certain articles of diet should be insisted on or should be prohibited; but to teach patients, as Dr. Joslin would teach them, to estimate the number of calories in their diet, and to regulate the proportions of protein, fat, and starch, seems to us very unwise and very undesirable.

In the first place, we do not know within a very wide margin what number of calories are necessary to sustain the body in health. Different authorities make extraordinarily different estimates. In the second place, what is treated of is hospital dietetics, and the ordinary estimates of the proper number of calories, even if these estimates agreed or were within measurable distance of one another, do not apply to patients in hospitals. It is ridiculous to suppose that a man lying all day in bed, amply clothed, in a well-warmed ward, and taking no exercise, requires the same number of calories in his food as he would require if he were doing laborious work in the open air, thinly clad, and in a bitter east wind. It is almost as ridiculous to suppose that the undersized City clerk weighing eight or nine stone needs as many calories as the hulking navvy of twice his weight. The relative requirements of the body as to protein, fat, and starch can be estimated only to a very vague approximation, and the relative proportion which is right for one man in one set of circumstances may be very wrong indeed for another man, or for the same man in different circumstances. No doubt the ultimate aim of science in all things is to weigh and measure, and to reduce our knowledge to numerical exactitude, but nothing could be more misleading than to attempt measurement prematurely; nothing more pernicious in practice than to take as accurate figures that are in any case but a vague approximation, and even as a vague approximation are true only for certain special cases. There never was a case in which a little knowledge was more dangerous.

Moreover, even supposing that we could calculate with accuracy the number of calories necessary in any given case, and the strict proportion of protein, fat, and starch which is undoubtedly the best in that case, and feed the patient accordingly, we might easily kill the patient with an inappropriate diet. The discovery of vitamines shows that the number of calories is not everything, and that even when we have added to the correct number of calories the correct proportion by weight of the several ingredients, there still remains an ingredient that is vitally necessary, but of whose nature we are ignorant, and of whose proper proportion in the diet we are ignorant. This ought to make us hesitate to prescribe with dogmatic certainty a so-called scientific dietary. For aught we know there may be other ingredients equally elusive, and equally necessary to a healthy diet.

It is probable that there is as much humbug and as much self-deception in the matter of diet as in any other department of medicine, even including the functions of the liver. It is common to speak of milky puddings as "light diet," and to consider that they are easily digestible and appropriate in almost every form of disease, especially of acute disease. Common experience shows that to some persons they are indigestible, and recent researches have shown that in some cases they are positively poisonous.

The attempt to estimate accurately things that have not been hitherto estimated accurately is altogether laudable; but the pretence that we have estimated a thing accurately when we have concocted a set of very questionable statistics is not science—it is quackery. After all, the human race has managed to survive for a good many years without its members weighing and measuring all the food they ate, or even estimating the due proportions of its ingredients in preparing their meals. Our prehistoric forefathers no more knew that it was necessary to consume so many calories, or so much protein, than we knew a few years ago that it was necessary to consume vitamines. Yet they lived, and many of them lived long and healthy lives by merely following their instinct and eating what they had an appetite for. The experience of hundreds of thousands of years should not be lightly thrown away in a moment of enthusiasm produced by very imperfect knowledge. If food is appetising, the *primâ facie* presumption is that it is wholesome. This presumption may be rebutted, but until it is rebutted it is pretty safe to act upon it. Of course it may be that even appetising food, because of its very appetising quality, is unsuitable to certain persons. It is unsuitable to those, for instance, who habitually over-eat. Such persons are better with unappetising food. But, broadly and generally, it is safe to assume that the materials and proportions and quantities of food that have proved nourishing and satisfactory for thousands of generations have not suddenly lost their nourishing and satisfactory character, and that before we alter either the materials, the proportions, or the quantities we should have very sure ground to go upon. Above all, sudden radical changes are to be deprecated. Even a diet that is ideally the best is not necessarily the best for a man who has long been accustomed to thrive upon a very different diet, and up to the present no two authorities agree upon the diet that is ideally the best.

